# Dexmedetomidine-Induced Fever in a 66-Year-Old Male With Pneumonia and Pleural Effusion

**DOI:** 10.7759/cureus.26529

**Published:** 2022-07-03

**Authors:** Ayesha Imtiaz, Ankita Prasad, Andrea Marin, Lwoodsky Charles, Varun Vankeshwaram, Pramil Cheriyath

**Affiliations:** 1 Internal Medicine, Hackensack Meridian Health, Ocean Medical Center, Brick, USA; 2 Internal Medicine, Ocean University Medical Center, Brick, USA; 3 Medicine, Zaporozhye State Medical University, Zaporozhye, UKR

**Keywords:** dexmedetomidine, adverse effect, ventilated, sedation, naranjo scale, drug fever

## Abstract

Drug-induced fever is a significant adverse effect as many commonly used medications can cause this. The incidence of drug fever is even higher in critical care settings because multiple medications are being administered simultaneously. This poses a serious problem in critical care settings as any new fever in these settings also implies any new infection or worsening of preexisting conditions. This may lead to a detailed investigation for the cause of fever, which can be time-consuming, invasive, costly, and may also increase the duration of stay along with an associated increase in morbidity and mortality. We want to highlight an adverse drug event through a documented case of *Dexmedetomidine-*induced fever in a critical care patient with multiple pathologies.

## Introduction

Drug-induced fevers account for 3-5% of all adverse drug reactions [1]. The body's core temperature is highly regulated to maintain core temperatures between 35.3°F to 37.7°F The hypothalamus moderates the body's fever response to pyrogens, interleukins, interferons, hypersensitivity, and idiosyncratic reactions. Drug-induced fever is a febrile response to a drug associated with starting the offending medication and wanes with the discontinuation of the medicine. Drug-induced fever is usually a diagnosis of exclusion [2]. The causes of drug fever are many, like hypersensitivity to the medication, pharmacologic extension of the drug's mechanism of action, contamination of the IV fluids or medications, pyrogens present in the infusion sets, and pyrogenic properties of drugs, inflammation of the vein used for IV medication. The most common drugs that cause fever are antimicrobials, anticonvulsants, heparin, allopurinol, and biologicals.

Dexmedetomidine is a highly selective alpha-2 adrenergic receptor agonist and a sympatholytic used for light to medium sedation as an IV infusion in the intensive care unit (ICU). Its distribution half-life is six minutes, and elimination half-life is two hours, and it crosses the blood-brain barrier. Sedation with dexmedetomidine is widely used in ventilated patients as it is not associated with respiratory depression like many other sedatives and may help ventilated patients' early weaning and extubation. It is beneficial in delirious, combative, or uncooperative patients, and its effects resemble natural deep sleep after sleep deprivation [3]. Dexmedetomidine causes sedation and sleep-producing action through central pre-and postsynaptic α2-receptors in the locus coerulus [4-5]. There are multiple cases of drug-induced fevers caused by dexmedetomidine infusion in critical care patients. Fever with dexmedetomidine is supposed to be caused by an allergic reaction. The FDA as well as the dexmedetomidine labels list fever and hyperpyrexia as side effects. 

## Case presentation

**Figure 1:**
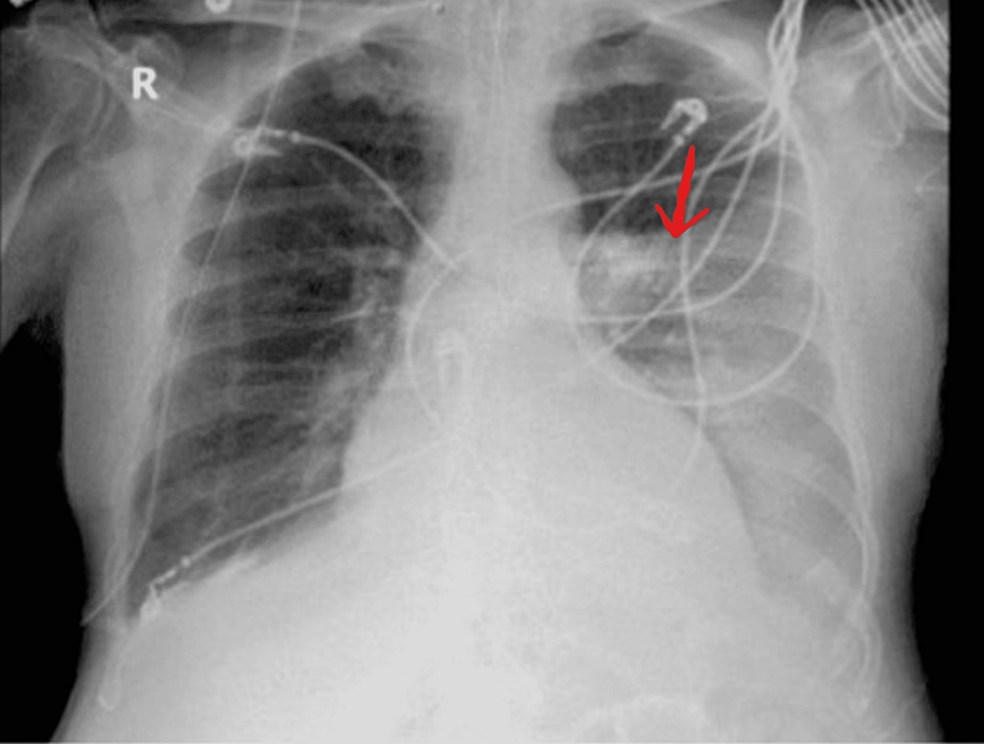
Chest x-ray showing left-sided pleural effusion (red arrow)

A 66-year-old-male presented to the hospital with shortness of breath for a few days, which had worsened overnight. He had received IV magnesium, methylprednisolone, lorazepam, and albuterol nebulization by emergency medical technicians (EMT) before arriving in the emergency room (ER). His oxygen saturation was in the low 70s on room air, and he was placed on a nonrebreather mask, which was changed to BiPAP by the EMTs on the way to ER. He had a pertinent history of chronic obstructive pulmonary disease (not on home oxygen) and chronic heart failure. At presentation, he was agitated and tachypneic with a heart rate of 124/minute, blood pressure of 91/67mm Hg, and SpO2 of 74% with generalized rhonchi and decreased air entry on the left lower chest physical exam. Blood gas showed hypercapnic respiratory failure with acidosis with a pH of 7.1 and pCO2 of 71 secondary to a COPD exacerbation (see Table 1 for the remaining laboratory findings). The patient was somnolent and apneic on BiPAP and was intubated and transferred to the intensive care unit for further management. Chest imaging showed moderate pleural effusions with atelectasis, patchy bilateral ground-glass infiltrates, and no pulmonary embolism (Figure 1).

**Table 1:** Laboratory findings at presentation

Investigations	Result	Normal Values
Complete blood Count(CBC)	15.3 x 10*3/ uL (H)	4.5-11x 10*3/ul
Hemoglobin	11.1g/dl	12-17.5g/dl
Platelet count	330 x 10*3/ul	150-450 x 10*3/ul
Neutrophils	72.5%(H)	50-70%
Lymphocytes	16.3(L)	25-43%
Immature Granulocytes	1.4	0.0-1.5%
Sodium	132(L) mmol/L	136-145 mmol/L
Potassium	5.4(H) mmol/L	3.5-5.2 mmol/L
Chloride	99 mmol/L	96-110 mmol/L
Blood urea nitrogen	13 mg/dl	5-25 mg/dl
Creatinine	1.05 mg/dl	0.61-1.24 mg/dl
Calcium	8.4 mg/dl	8.5-10.5 mg/dl
Albumin	3.5 g/dl	3.5-5.0 g/dl
Total protein	6.6 g/dl	6.0-8.0 g/dl
Alkaline phosphatase	88 U/L	38-126 U/L
Alanine transferase	41 U/L	10-60 U/L
Aspartate transferase	47 U/L	10-42 U/L
total Bilirubin	0.8 mg/dl	0.0-1.3 mg/dl
Glucose	486 mg/dl	70-99 mg/dl
Anion Gap	14 mmol/L	5-13 mmol/L
Lactate	2.5 mmol/L	0.5-2.0 mmol/L
Magnesium	3.6 mg/dl	1.3-2.5 mg/dl
Troponin	0.04 ng/ml	<0.04 ng/ml
Prothrombin time	10.9 SEC	10.0-13.1
INR	0.96	0.88-1.15
Brain Natriuretic peptide	550	<100
SARS CoV (PCR)	Negative	
Sputum (gram Stain and culture)	Many Candida Albicans and Klebsiella variioli	
Pleural Fluid culture	Staphylococcus epidermidis	

Dexmedetomidine was started early during the admission at 0.3mcg and was gradually increased to 1.2mcg. He developed atrial flutter with rapid ventricular rate and was started on IV diltiazem, successfully controlling his heart rate. He had an echocardiogram to assess left ventricular function, which showed a reduced ejection fraction (21-25%) but no vegetations. A left-sided chest tube was placed for pleural effusion; it has significant drainage (more than 700 ml) and results in the subsequent improvement of oxygen requirement on the ventilator. Pleural fluid cultures grew *Staphylococcus epidermidis.* The sputum culture was positive for *Candida albicans* plus *Klebsiella. *His white cell counts and neutrophilia improved along with inflammatory markers like C-reactive protein. Despite marked improvement in respiratory status and lowering of inflammatory markers, the patient started developing fevers on Day 3 of admission. Repeat blood and urine cultures did not show any growth, and sputum grew a few yeasts and gram-positive cocci, considered nonsignificant and a part of normal flora. The patient had four blood cultures sent during this time, all of which were negative. He was started on IV ceftriaxone and azithromycin at admission, which were later changed on the fourth day to IV piprecillin/tazobactum in view of fever. 

As he continued to be persistently febrile every night, a respiratory panel was sent, which also came back negative. His medications were then reassessed, and it was found that his fevers had a temporal association with dexmedetomidine introduction, and the fever response was dose-dependent on its infusion rate. Given the temporal association and no other identifiable cause of fever, dexmedetomidine was suspected as the cause of fever and discontinued. His sedation was switched to propofol infusion, and the patient had defervescence in the next four hours. He remained afebrile for the rest of his course after discontinuation of dexmedetomidine. After a weaning trial, the patient was successfully extubated. His chest tube was removed without complications, and a 2D echocardiogram was repeated, which showed an improved ejection fraction to 45%. He was later discharged in a stable condition and instructed to follow up as an outpatient with his pulmonologist and cardiologist.

## Discussion

There have been many documented cases of dexmedetomidine-induced fever before this. This is the biggest pitfall of its usage. While it is phenomenal for regular usage, it can cause drug fever that we can fail to correlate with early on in a ventilated patient. Our patient was already receiving antibiotics for pneumonia and pleural effusion, and there was a chance of antibiotics failure and/or acquiring a nosocomial infection as well as many other causes of fever. When faced with such a situation, we usually consider the probability of drug fever as the last thing after we have exhausted all other differentials. This leads to a series of investigations, delays the diagnosis, and increases the ICU stay, which has its own set of problems. Similar to our case are two case reports that implicate dexmedetomidine as the cause of fever one by Okabe et al. (2009) [6] and another by Reeve and Cooper (2013) [7]. Okabe and colleagues were the first to describe dexmedetomidine-induced fever. In their case, the fever started 22 hours after an infusion of 0.5-0.7 µg per kg per hour. They stopped the medication for a while to assist in extubation. However, on resuming the infusion, fevers continued spiking up to 40.6°C. Discontinuing dexmedetomidine stopped the fever approximately four hours later. They ruled out all other causes of fever. A similar pattern was recognized in all other studies, i.e., the temporal association with the start and fevers dissipating about four to five hours after stopping of infusion, thorough investigations ruling out any other causes of fever, and pyrexia unresponsive to fever control measures, even to dantrolene which was used in the study by Okabe et al. [6,7]. These authors assigned their case a Naranjo score of 3 (‘possible’) [2]. Drug-induced fever starts within a few days of beginning dexmedetomidine and dissipates entirely in a 3-5 half-life of elimination [8]. It has a distribution half-life of six minutes and an elimination half-life of two hours [8]. In ICU patients, the half-life of dexmedetomidine elimination is between 2.2 and 3.7 hours [9]. This correlates well with the end of fever three to four hours after stopping the infusion, as in other studies.

Sedation with dexmedetomidine helps wean patients off the ventilator and extubate earlier than other sedatives. A Cochrane review covering seven studies and 1624 participants compared dexmedetomidine in ICU sedation with traditional sedatives [10]. It reduced the duration of mechanical ventilation by 22% and the length of ICU stay by 14% [10]. These make it an attractive sedation option for ICU patients. The FDA as well as the dexmedetomidine label from the year 1999 list fever and hyperpyrexia as side effects [11]. Adverse Drug Reaction (ADR) Probability Scale, often referred to as the Naranjo Scale, can be used to diagnose drug-induced fever [2,12]. The Adverse Drug Reaction Scale consists of 10 questions answered as either yes, no, or “Do not know” and points (-1, 0, +1 or +2) are given for each answer. Total scores range from -4 to +13; definite if the score is nine or higher, probable if 5 to 8, possible if 1 to 4, and doubtful if 0 or less (see table in appendix). 

## Conclusions

It is essential to consider drug-induced fevers with unremitting fevers and the temporal association when starting fever-causing medications like dexmedetomidine in ICU. Considering the advantages of dexmedetomidine over propofol and benzodiazepines for sedation and its widespread use in ICU, it is necessary to remember this adverse effect to avoid unnecessary invasive and expensive testing and treatment and avoid a more extended ICU stay. It will help bring down morbidity and healthcare costs if we can formulate guidelines where we would first stop dexmedetomidine and then proceed with workup for fever instead of going the other way around and considering it as a cause of fever after we have exhausted all other diagnoses. This case is a reminder to restructure guidelines for fever management in critical care for a better quality of care.

## Appendices

Table 2 shows the Adverse Drug Reaction (ADR) Probability Scale, often referred to as the Naranjo Scale. 

**Table 2:** Naranjo Scale (Adverse Drug Reaction Scale)

Q.No.	Question	Response (Yes/No/Do Not Know)	Score
1	Are there previous conclusive reports of this reaction?		
2	Did the adverse event appear after the drug was given?		
3	Did the adverse reaction improve when the drug was discontinued, or a specific antagonist was given?		
4	Did the adverse reaction reappear upon readministering the drug?		
5	Were there other possible causes for the reaction?		
6	Did the adverse reaction reappear upon administration of a placebo?		
7	Was the drug detected in toxic concentrations in the blood or other fluids?		
8	Was the reaction worsened upon increasing the dose? Or was the reaction lessened upon decreasing the dose?		
9	Did the patient have a similar reaction to the drug or a related agent in the past?		
10	Was the adverse event confirmed by any other objective evidence?		
	Total		
